# The nPYc-Toolbox, a Python module for the pre-processing, quality-control and analysis of metabolic profiling datasets

**DOI:** 10.1093/bioinformatics/btz566

**Published:** 2019-07-27

**Authors:** Caroline J Sands, Arnaud M Wolfer, Gonçalo D S Correia, Noureddin Sadawi, Arfan Ahmed, Beatriz Jiménez, Matthew R Lewis, Robert C Glen, Jeremy K Nicholson, Jake T M Pearce

**Affiliations:** 1 National Phenome Centre and Imperial Clinical Phenotyping Centre, Department of Surgery & Cancer, Imperial College London, London, UK; 2 Division of Integrative Systems Medicine and Digestive Diseases, Department of Surgery & Cancer, Imperial College London, South Kensington, London, UK

## Abstract

**Summary:**

As large-scale metabolic phenotyping studies become increasingly common, the need for systemic methods for pre-processing and quality control (QC) of analytical data prior to statistical analysis has become increasingly important, both within a study, and to allow meaningful inter-study comparisons. The nPYc-Toolbox provides software for the import, pre-processing, QC and visualization of metabolic phenotyping datasets, either interactively, or in automated pipelines.

**Availability and implementation:**

The nPYc-Toolbox is implemented in Python, and is freely available from the Python package index https://pypi.org/project/nPYc/, source is available at https://github.com/phenomecentre/nPYc-Toolbox. Full documentation can be found at http://npyc-toolbox.readthedocs.io/ and exemplar datasets and tutorials at https://github.com/phenomecentre/nPYc-toolbox-tutorials.

## 1 Introduction

Metabolic phenotyping offers a powerful window into gene-environment interactions (Nicholson *et al.*, 2012). Inter-study comparison in the field is complicated by the diversity of analytical platforms used to generate data, and the lack of standard quality criteria. Standards are emerging around the most common platforms: Nuclear Magnetic Resonance spectroscopy (NMR), and hyphenated-Mass Spectrometry (MS), and procedures for the acquisition of profiles from human biofluid samples in particular are well established (Dona *et al.*, 2014; Lewis *et al.*, 2016). However, QC in profiling studies has typically been conducted on an ad-hoc basis in individual studies, although there is an increasing push towards the systematization and automation of pre-processing procedures (Giacomoni *et al.*, 2015; van Rijswijk *et al.*, 2017).

**Fig. 1.**
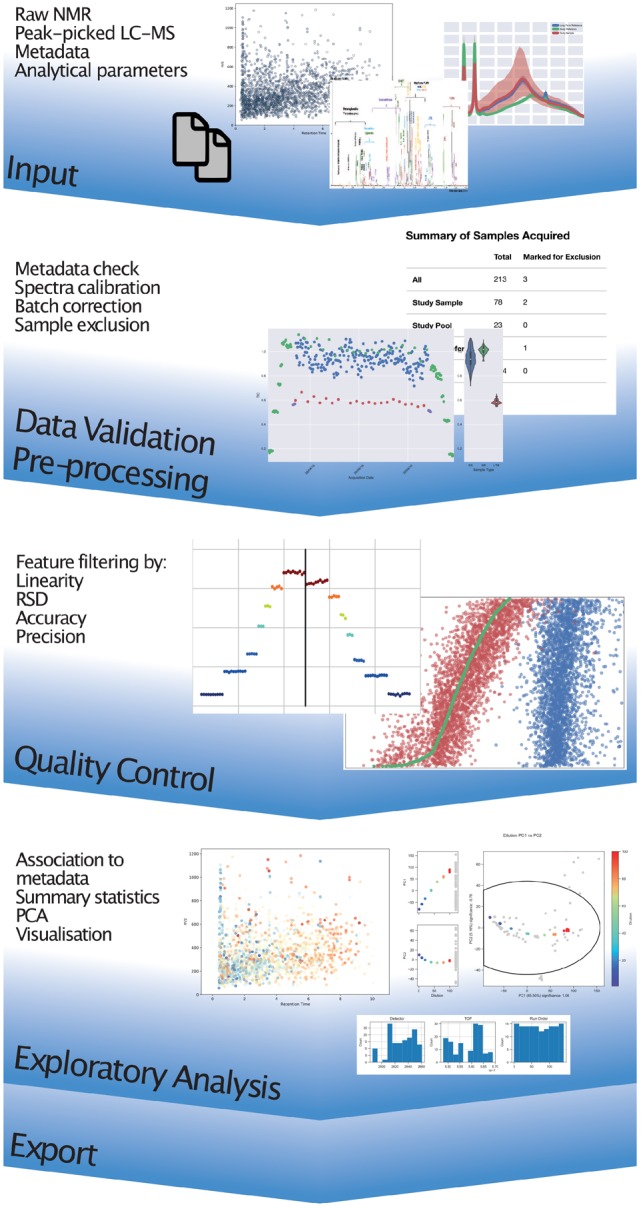
Conceptual diagram outlining the workflow embodied by the toolbox, from import of raw or feature-extracted datasets, preprocessing and filtering, QC, visualization and export

The toolbox presented here provides software for pre-processing, QC and visualization of metabolic profiling datasets, embodying the MRC-NIHR National Phenome Centre (NPC) practices and focusing on the interpretability of the output to both data generators and analysts (Fig. 1).

## 2 The nPYc-Toolbox

### 2.1 Implementation

The toolbox is designed to allow reproducible processing of datasets with minimal reliance on human judgement during the process. It may be used interactively (e.g. in a Jupyter notebook, for which tutorials are provided), or as an API in automated workflows. It is coded in Python 3.6. To account for the differing processing workflows expected of the common analytical datasets outlined above, the toolbox subclasses its *Dataset* object; the *NMRDataset* encapsulates methods for handling spectral NMR data; *MSDataset* for discretely measured (peak-picked) hyphenated-MS profiling datasets; and *TargetedDataset* for targeted, quantified datasets, derived from MS, NMR or any other analytical platform.

### 2.2 Features

Dataset objects are initialized from raw (Bruker NMR) or feature-extracted data [outputs of software such as XCMS (Tautenhahn *et al.*, 2008), Progenesis QI^TM^, TargetLynx^TM^, &c], and associated with study design parameters or metadata read directly from the raw data or from csv files. The csv template is structured so that each row corresponds to a sample, and columns contain a set of mandatory fields, and any other user required metadata. The role that each sample plays in the assay and its pre-processing is delineated using a standardized nomenclature.

Routines for pre-processing 1D NMR spectra by the automated calculation of QC metrics assessing line-width, water suppression and baseline stability are implemented (as described by Dona *et al*., 2014). Current best-practices in QC of profiling LC-MS (Broadhurst *et al.*, 2018; Dunn *et al.*, 2011; Lewis *et al.*, 2016; Want *et al.*, 2010) include repeated injections of pooled quality control samples, and a serial dilution of the reference sample to calculate *per* feature analytical precision and linearity of response. Correction of run-order effects follows an adapted version of the LOWESS approach proposed by Dunn *et al.* (2011). The targeted pre-processing module contains a set of reports and data consistency checks, to assist analysts in assessing the presence of batch effects, standardizing the linearity range over multiple batches, and visualizing the distribution ranges of samples assayed and relationships within the limits of quantification.

Exploratory data analysis with PCA is used to assess the impact of the QC choices on the final dataset, and screen for associations between acquisition parameters and study factors.

Parameter sets can be specified as JSON dictionaries, allowing simple automation and generation of standardized workflows with basic auditing of all manipulations in a dataset. This toolbox can therefore be used to ensure reproducible pre-processing and quality control. Processed datasets can be exported as csv files in a number of different formats.

## 3 Conclusion

The nPYc-Toolbox supports both profiling and targeted metabolic phenotyping datasets, and provides tools for pre-processing, quality control and visualization.

## Funding

This work was supported by the Medical Research Council and National Institute for Health Research [grant number MC_PC_12025] through funding for the MRC-NIHR National Phenome Centre, infrastructure support was provided by the NIHR Imperial Biomedical Research Centre and PhenoMeNal, European Commission Horizon2020 programme, grant agreement number 654241.

*Conflict of Interest*: none declared.
